# Donanemab in early symptomatic Alzheimer’s disease: results from the TRAILBLAZER-ALZ 2 long-term extension

**DOI:** 10.1016/j.tjpad.2025.100446

**Published:** 2025-12-01

**Authors:** Jennifer A. Zimmer, John R. Sims, Cynthia D. Evans, Emel Serap Monkul Nery, Hong Wang, Alette M. Wessels, Giulia Tronchin, Shoichiro Sato, Lars Lau Raket, Scott W. Andersen, Christophe Sapin, Marie-Ange Paget, Ivelina Gueorguieva, Paul Ardayfio, Rashna Khanna, Dawn A. Brooks, Brandy R. Matthews, Mark A. Mintun

**Affiliations:** Eli Lilly and Company, Indianapolis, IN, USA

**Keywords:** Clinical benefit, Donanemab, Early symptomatic Alzheimer’s disease, Long-term extension data, TRAILBLAZER-ALZ 2

## Abstract

**Background:**

Donanemab significantly slowed clinical progression in participants with early symptomatic Alzheimer’s disease (AD) during the 76-week placebo-controlled period of TRAILBLAZER-ALZ 2.

**Methods:**

Participants who completed the placebo-controlled period were eligible for the 78-week, double-blind, long-term extension (LTE). Early-start participants were randomized to donanemab in the placebo-controlled period. Delayed-start participants (randomized to placebo) started donanemab in the LTE. Participants who met amyloid treatment course completion criteria were switched to placebo. An external control cohort comprised participants from the AD Neuroimaging Initiative (ADNI).

**Results:**

At 3 years, donanemab slowed disease progression on the Clinical Dementia Rating Scale (CDR)-Sum of Boxes (CDR-SB) in early-start participants versus a weighted ADNI control (-1.2 points; 95 % confidence interval [CI], -1.7, -0.7). Seventy-six weeks after initiating donanemab, delayed-start participants also demonstrated slower CDR-SB progression versus a weighted ADNI control (-0.8 points; 95 % CI, -1.3, -0.3). Participants who completed treatment by 52 weeks demonstrated similar slowing of CDR-SB progression at 3 years. Compared with delayed-start participants, early-start participants demonstrated a significantly lower risk of disease progression on the CDR-Global over 3 years (hazard ratio=0.73; p < 0.001). In both groups, >75 % of participants assessed by positron emission tomography 76 weeks after starting donanemab achieved amyloid clearance (<24.1 Centiloids). The addition of LTE data to prior modeling predicted a median reaccumulation rate of 2.4 Centiloids/year. No new safety signals were observed compared to the established donanemab safety profile.

**Conclusions:**

Over 3 years, donanemab-treated participants with early symptomatic AD demonstrated increasing clinical benefits and a consistent safety profile, with limited-duration dosing.

**Trial registration:**

ClinicalTrials.gov identifier NCT04437511

## Highlights

• Donanemab-treated participants showed increasing clinical benefits over 3 years.
• Clinical benefits of donanemab continued after the treatment course was completed.
• Amyloid reaccumulation was comparable to the natural amyloid accumulation rate.
• No new safety signals were seen versus the established safety profile of donanemab.

## Graphical abstract

**Figure:**
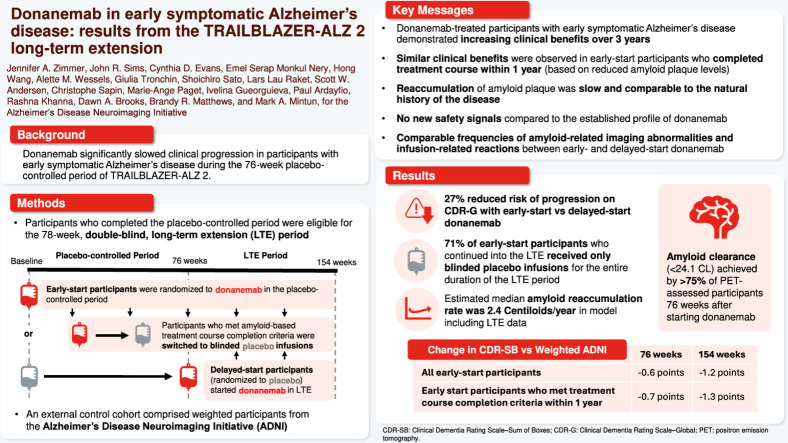

## 1 Introduction

Alzheimer’s disease (AD) is an age-related neurodegenerative disorder characterized by the accumulation of β-amyloid plaques and neurofibrillary tangles in the brain that cause progressive cognitive decline [1]. Randomized controlled clinical trials have shown that sufficient removal of amyloid plaque can slow disease progression [2,3]. In the pivotal phase 3 TRAILBLAZER-ALZ 2 trial, donanemab significantly slowed clinical progression compared with placebo at 76 weeks in participants with early symptomatic AD and amyloid and tau pathology. A distinct feature of this trial was the use of limited-duration dosing in which participants were switched to blinded placebo infusions after meeting treatment course completion criteria based on reduced amyloid plaque levels. Treatment course completion criteria were implemented to eliminate potentially unnecessary treatments and reduce cost for future patients.

Participants who completed the 76-week, placebo-controlled period of TRAILBLAZER-ALZ 2 were eligible to enter a participant- and investigator-blinded long-term extension (LTE) period, lasting an additional 78 weeks. The LTE provided an opportunity for participants initially randomized to placebo to receive blinded donanemab treatment if they entered the LTE period. Importantly, the LTE also included participants initially randomized to receive donanemab who met treatment course completion criteria during the placebo-controlled period, were switched to blinded placebo infusions, and remained on blinded placebo infusions. Finally, participants who did not meet treatment course completion criteria continued to receive donanemab treatment during the LTE period. Here, we report the results from the LTE period of TRAILBLAZER-ALZ 2, which was designed to evaluate the long-term efficacy and safety of donanemab after limited-duration dosing.

## 2 Methods

### 2.1 Trial design

The study design for the placebo-controlled period of TRAILBLAZER-ALZ 2 was previously described [2]. Briefly, TRAILBLAZER-ALZ 2 is a 76-week, phase 3, randomized, double-blind, parallel-group, multicenter, placebo-controlled trial with an additional 78-week LTE period (ClinicalTrials.gov identifier: NCT04437511). Eligible participants were randomly assigned in a 1:1 ratio to receive donanemab (700 mg for the first three doses and 1400 mg thereafter) or placebo, administered intravenously every 4 weeks. During the placebo-controlled period, participants’ treatment assignments were switched in a blinded procedure from donanemab to placebo at week 24 or 52 if the participant met treatment course completion criteria, defined as an amyloid plaque level of <11 Centiloids (CL) on any single positron emission tomography (PET) scan or <25 CL on two consecutive PET scans.

**Fig. 1:**
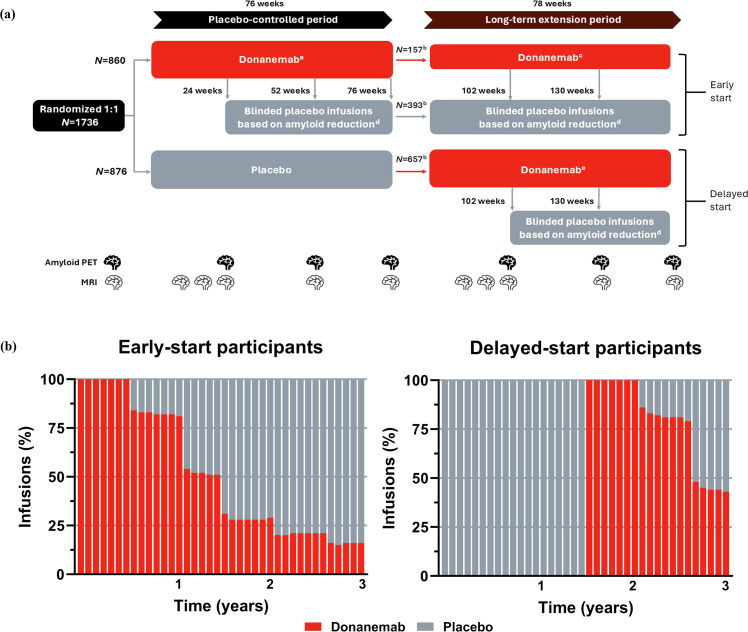
TRAILBLAZER-ALZ 2 overview: (a) study design and (b) study dosing. (a) The study design of TRAILBLAZER-ALZ 2 allowed for participants receiving donanemab to switch to blinded placebo infusions at 24, 52, 76, 102, or 130 weeks if they met treatment course completion criteria based on amyloid PET. (b) The percentage of donanemab infusions in participants randomized to donanemab (ie, early-start group) continued to decline as treatment course completion criteria based on amyloid PET were met. A similar trend was observed among participants randomized to placebo (ie, delayed-start group) who switched to donanemab during the LTE period. ^a^ Donanemab was administered intravenously at 700 mg Q4W for the first three doses and 1400 mg Q4W thereafter; ^b^ Number of participants receiving at least one infusion in the LTE period; ^c^ Participants randomized to donanemab during the PC period who did not meet the treatment course completion criteria by 76 weeks continued receiving donanemab Q4W; ^d^ Participants who met prespecified treatment course completion criteria based on amyloid PET were switched in a blinded fashion to placebo Q4W (saline infusion); ^e^ Participants randomized to placebo Q4W during the PC period were assigned to receive donanemab Q4W starting 78 weeks after randomization and followed the same dose-titration schedule as participants during the PC period. LTE: long-term extension; MRI: magnetic resonance imaging; N: number of participants; PC: placebo-controlled; PET: positron emission tomography; Q4W: every 4 weeks. Copyright © 2025 Eli Lilly and Company. All rights reserved. Permission for any use should be sought from Eli Lilly and Company.

At the end of the 76-week placebo-controlled period, participants randomized to donanemab who had not met treatment course completion criteria continued to receive donanemab in the 78-week LTE period. Participants randomized to receive placebo during the placebo-controlled period who remained in the study were assigned to receive donanemab at the start of the LTE period. These participants followed the same dose-titration schedule as participants treated with donanemab during the placebo-controlled period. The treatment assignment for any participants who received donanemab during the LTE period was switched to placebo in a blinded procedure if the participant met the previously described treatment course completion criteria at week 102 or 130. Participants and investigators remained blinded to treatment assignments throughout the LTE period. Fig. 1a illustrates the trial design for TRAILBLAZER-ALZ 2.

When describing the LTE period, the “early-start” group refers to participants who were initially randomized to receive donanemab. The early-start group also included participants whose treatment assignments were switched to blinded placebo infusions if they met treatment course completion criteria based on reduced amyloid levels at week 24, 52, 76, 102, or 130. The “delayed-start” group refers to participants who were initially randomized to receive placebo, continued into the LTE period, and started donanemab treatment in the LTE. The delayed-start group also included participants whose treatment assignments were switched to blinded placebo infusions if they met treatment course completion criteria based on reduced amyloid levels at week 102 or 130.

TRAILBLAZER-ALZ 2 was conducted at 277 centers in eight countries, including Australia, Canada, the Czech Republic, Japan, the Netherlands, Poland, the United Kingdom, and the United States. All procedures were performed in compliance with relevant laws and institutional guidelines and have been approved by the appropriate institutional committees. The privacy rights of all participants were observed. All participants and their study partners provided written informed consent.

### 2.2 Participants

Participants from the placebo-controlled period of the study were not required to meet additional eligibility criteria to enter the LTE period. Eligible participants for entry into the placebo-controlled period of the study were 60 to 85 years of age with a screening Mini-Mental State Examination (MMSE) score of 20 to 28, amyloid pathology (≥37 CL) assessed with ^18^F-florbetapir or ^18^F-florbetaben PET scans, and presence of tau pathology assessed by ^18^F-flortaucipir PET imaging with central image evaluation. Key exclusion criteria included the presence of amyloid-related imaging abnormalities (ARIA) of edema/effusion (ARIA-E), more than four cerebral microhemorrhages, more than one area of superficial siderosis, and any intracerebral hemorrhage >1 cm or severe white matter disease shown on magnetic resonance imaging (MRI). The full eligibility criteria were previously reported [2].

### 2.3 External ADNI control cohort

The LTE of TRAILBLAZER-ALZ 2 does not include an internal placebo control to allow for comparison of disease progression in participants without amyloid-targeting treatment. Therefore, clinical changes assessed by the Clinical Dementia Rating Scale (CDR)–Sum of Boxes (CDR-SB) score were compared to a control cohort obtained from the Alzheimer’s Disease Neuroimaging Initiative (ADNI) database (https://adni.loni.usc.edu). Participants in the external ADNI data set generally represent an amyloid-targeting therapy–naïve AD population that, when matched to TRAILBLAZER-ALZ 2 study participants, can be used as a basis for comparison. ADNI participants were selected to meet the following clinical and biomarker criteria: presence of cognitive impairment and cerebrospinal fluid total tau-by-amyloid β42 ratio >0.28 (established criterion for amyloid pathology) [4].

The ADNI was launched in 2003 as a public-private partnership, led by principal investigator Michael W. Weiner, MD. The original goal of the ADNI was to test whether serial MRI, PET, other biological markers, and clinical and neuropsychological assessment can be combined to measure the progression of mild cognitive impairment and early AD. The current goals include validating biomarkers for clinical trials, improving the generalizability of ADNI data by increasing diversity in the participant cohort, and providing data concerning the diagnosis and progression of AD to the scientific community. For up-to-date information, see https://adni.loni.usc.edu.

### 2.4 Outcomes

The primary outcomes for TRAILBLAZER-ALZ 2 at 76 weeks were previously reported [2]. In the LTE, efficacy was evaluated using the least-squares (LS) mean change from baseline in the CDR-SB score. CDR-SB data were used for comparison to the external cohort due to their availability in the ADNI database. The CDR measures both cognition and function. CDR-SB total scores range from 0 to 18, with higher scores indicating greater impairment. The CDR–Global (CDR-G) score was used to analyze the risk of progressing to the next stage of disease between the early-start and delayed-start donanemab treatment groups. CDR-G total scores range from 0 (no dementia) to 3 (severe dementia), with higher scores indicating greater impairment.

Reported biomarkers included amyloid plaque reduction and clearance at weeks 102, 130, and 154. Amyloid reaccumulation was assessed in participants who met treatment course completion criteria by 52 weeks. The overall reaccumulation rate was estimated using data from the present study combined with data from three additional donanemab studies [5]. Relevant safety assessments included adverse event (AE) reporting and centrally read MRI scans.

### 2.5 Statistical analysis

All analyses were exploratory and not controlled for multiplicity.

#### 2.5.1 Efficacy

Efficacy in the LTE period was evaluated independently for the early-start group and the delayed-start group. A propensity score weighting method was used to ensure that baseline disease characteristics of the TRAILBLAZER-ALZ 2 groups were balanced with the external control population from ADNI. The TRAILBLAZER-ALZ 2 reference populations used for each weighting procedure were (1) participants in the placebo arm and (2) the subset of participants receiving placebo who entered the LTE, when comparing to the delayed-start group. Propensity score weights were estimated for the ADNI cohort with the average treatment effect among the treated estimand using the inverse probability weighting method from the generalized linear model of the WeightIt R package [6,7]. The weighting procedure employed the following baseline covariates: age, sex, apolipoprotein E (*APOE*) ε4 status (noncarrier, heterozygous, or homozygous), CDR-SB score, 13-item cognitive subscale of the Alzheimer Disease Assessment Scale (ADAS-Cog_13_) score, and screening MMSE score. All common variables between the two data sources were adjusted for, apart from baseline amyloid CL level which was missing for 43 % of participants in the defined population pulled from the ADNI database. **Fig. S1** includes an unweighted sensitivity analysis of ADNI participants with amyloid pathology on PET (≥37 CL) and who were global tau PET positive.

Propensity weights were checked to ensure covariates were balanced (standardized mean difference of <0.10 for each covariate) and to assess extreme weights (trimmed to the 95th percentile for weights higher than the 95th percentile) [8] and the impact on the effective sample size. Finally, three analyses using the same mixed model for repeated measures (MMRM) approach were performed to examine change from baseline in the CDR-SB score. The first MMRM analysis used data from the TRAILBLAZER-ALZ 2 placebo arm and the corresponding weighted ADNI cohort up to 76 weeks to assess the progression of decline of the weighted ADNI cohort versus that of the TRAILBLAZER-ALZ 2 placebo arm. The second and third MMRM analyses were performed to assess the treatment difference at 154 weeks between the donanemab arm (early- and delayed-start group, respectively) in the TRAILBLAZER-ALZ 2 LTE and the respective weighted ADNI cohort. Covariates included treatment, age, *APOE* ε4 status, baseline CDR-SB score, baseline ADAS-Cog_13_ score, screening MMSE score, and visit, as well as visit-by-treatment and visit-by-baseline CDR-SB interaction terms. Treatment differences were computed as LS mean differences between the TRAILBLAZER-ALZ 2 groups and the external weighted ADNI control cohort at each visit. Differences in area under the curve (AUC) were computed as the LS mean difference between the AUCs, with weights derived using the trapezoidal rule. Relative time savings at the end of the LTE were estimated by calculating the ADNI cohort and early-start group lines between 24 and 36 months and then back-calculating the ADNI timepoint based on the mean of the 36-month early-start group.

Advancement to the next clinical stage was determined in an event-driven analysis between the early- and delayed-start groups and defined as an increase in the CDR-G score at two consecutive visits, where participants were assessed every 3 months for progression to the next stage of AD. The visit date of the first consecutive visit was used in the analysis. The hazard ratio, 95 % confidence interval (CI), and *p* value for change in the CDR-G score were calculated using a Cox proportional hazards model, stratified by pooled investigator and baseline tau level. The model included age, CDR-G score, and acetylcholinesterase inhibitor or memantine use as baseline covariates and group (early start or delayed start) as a fixed factor.

#### 2.5.2 Biomarkers

The LS mean, standard error, 95 % CI, and *p* value for change from baseline in amyloid PET CL were estimated using an MMRM with treatment, visit, and treatment-by-visit interaction as fixed factors, and score, score-by-visit interaction, age, and tau category as baseline covariates. Clearance was defined as achieving amyloid plaque levels <24.1 CL, which is largely consistent with a negative visual scan [9,10]. Amyloid reaccumulation over time was plotted using mean CL values for the early-start participants who met treatment course completion criteria by 52 weeks. Using previously reported methods [11,12], the amyloid reaccumulation rate was calculated by incorporating the LTE data from TRAILBLAZER-ALZ 2 into an existing exposure-response model of donanemab studies, which included a phase 1b study (NCT02624778), TRAILBLAZER-ALZ (phase 2; NCT03367403), TRAILBLAZER-EXT (phase 2 LTE parts B and C; NCT04640077), and TRAILBLAZER-ALZ 2 (phase 3; placebo-controlled portion and safety addendum; NCT04437511) [5].

#### 2.5.3 Safety

All safety outcomes are descriptive. Time to the first ARIA-E event was based on MRI or treatment-emergent AE (TEAE) cluster reporting. The timeframe for this analysis included the treatment period and extended to 57 days. To evaluate the risk for ARIA after donanemab dosing was completed, new ARIA events identified by MRI or new or worsening AEs were assessed at 6-month intervals. The first interval (months 0–6) started 58 days after the last donanemab dose. ARIA incidence was also analyzed in participants who met treatment course completion criteria by 52 weeks. Analysis of these participants includes the observation time-adjusted incidence rate (OAIR) to facilitate comparison between populations with differing amounts of observation time. The OAIR was calculated by multiplying the number of participants with at least one AE in the given category by 100 and dividing by the participant-years at risk.

## 3 Results

### 3.1 Participants

Among the 860 participants randomized to receive donanemab during the placebo-controlled period (ie, the early-start group), 550 (64.0 %) received at least one infusion in the LTE period. Of these participants, 393 had their treatment assignment switched to placebo at 24, 52, or 76 weeks, and 157 continued treatment with donanemab in the LTE (Fig. 1a). Of the 876 participants randomized to receive placebo during the placebo-controlled period, 657 (75.0 %) entered the LTE and had their treatment assignment switched to donanemab (ie, the delayed-start group). Among early-start participants who received at least one infusion in the LTE, 74.3 % of those who switched to placebo and 81.5 % of those who continued donanemab completed the LTE, respectively. Among delayed-start participants who received at least one infusion in the LTE, 72.3 % completed the LTE. The full study disposition of participants who received at least one infusion during the LTE period is shown in **Fig. S2**. By the end of the LTE, the majority of infusions administered in both the early- and delayed-start groups were placebo. Fig. 1b illustrates the percentage of donanemab infusions over time for each group.

Of the 2430 participants evaluated from the ADNI database for matching the TRAILBLAZER-ALZ 2 population, 534 met the eligibility criteria and were included in propensity score weighting calculations, yielding an effective sample size for analyses of 268 for the early-start group (**Fig. S3a**) and 301 for the delayed-start group (**Fig. S3b**). The covariate balance of propensity weights for both the early- and delayed-start groups is shown in **Fig. S4**. The external ADNI control cohort closely aligned with the trajectory of the placebo group, suggesting it was an appropriate control group to evaluate the treatment effect of donanemab (**Fig. S5**).

Demographics and disease characteristics at the start of the LTE period are presented in **Table S1** for early-start participants who met treatment course completion criteria and switched to placebo by 76 weeks (*N* = 393), early-start participants who continued donanemab (*N* = 157), and delayed-start participants (*N* = 657). Compared with early-start participants who continued to receive donanemab, a greater percentage of early-start participants who completed donanemab treatment by 76 weeks were female (58.3 % vs 51.0 %) and a smaller percentage were *APOE* ε4 homozygotes (11.5 % vs 25.5 %). Early-start participants who continued donanemab treatment in the LTE period as compared to early-start participants who completed donanemab treatment by 76 weeks showed a higher mean (standard deviation [SD]) amyloid level at study screening (122.0 [34.8] CL vs 94.8 [31.6] CL) and at the LTE baseline (37.5 [21.1] CL vs 3.7 [11.3] CL). Tau PET levels were similar across all treatment groups at the start of the LTE. Compared with the baseline disease characteristics previously published for the early-start participants (*N* = 860) [2], a greater percentage of delayed-start participants used symptomatic medication for AD (acetylcholinesterase inhibitor and/or memantine: 68.0 % vs 60.6 %) at the start of the LTE. MMSE scores for the delayed-start group at the start of the LTE also suggested greater cognitive impairment among these participants compared with scores for early-start participants at study baseline in the placebo-controlled period.

Baseline disease characteristics are also provided for the subset of early-start participants who met treatment course completion criteria by 52 weeks of the placebo-controlled period (**Table S2**). Compared with early-start participants overall (*N* = 860) [2], participants who completed treatment by 52 weeks had a lower mean (SD) amyloid level (90.8 [30.6] CL vs 103.5 [34.5] CL) and a higher mean MMSE total score (23.9 vs 22.4) at study baseline. A smaller percentage of early-start participants who met treatment course completion criteria by 52 weeks were *APOE* ε4 homozygotes compared with all early-start participants (9.5 % vs 16.7 %).

### 3.2 Efficacy

**Fig. 2:**
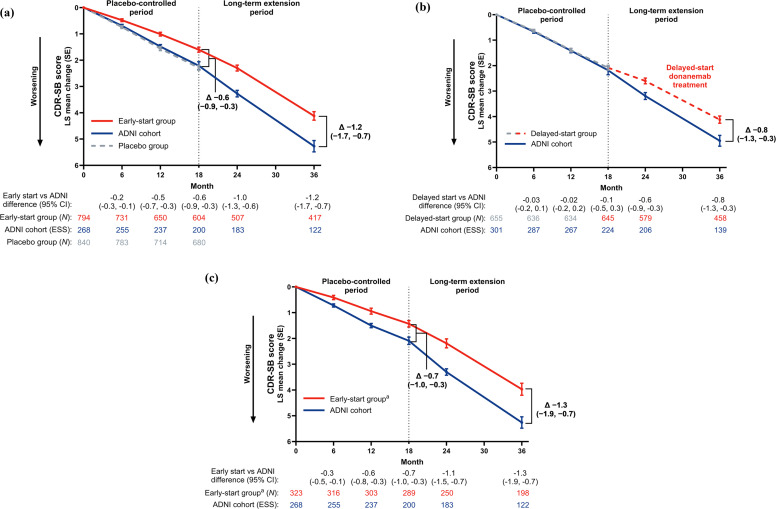
Clinical efficacy measured by change from baseline in the CDR-SB score. Efficacy was measured by change from baseline in the CDR-SB score versus the external weighted ADNI control cohort for participants in the (a) early-start group, (b) delayed-start group, and (c) early-start group who met treatment course completion criteria based on amyloid positron emission tomography by 52 weeks of the placebo-controlled period. Propensity score weights were estimated for the ADNI cohort with the average treatment effect among the treated estimand using the inverse probability weighting method from the generalized linear model. Change from baseline in the CDR-SB score was estimated using a mixed model for repeated measures using ADNI weights. ^a^ Includes only participants in the early-start group who met treatment course completion criteria by 52 weeks. ADNI: Alzheimer’s Disease Neuroimaging Initiative; CDR-SB: Clinical Dementia Rating Scale–Sum of Boxes; CI: confidence interval; ESS: effective sample size; LS: least-squares; N: number of participants; SE: standard error. Copyright © 2025 Eli Lilly and Company. All rights reserved. Permission for any use should be sought from Eli Lilly and Company.

**Fig. 3:**
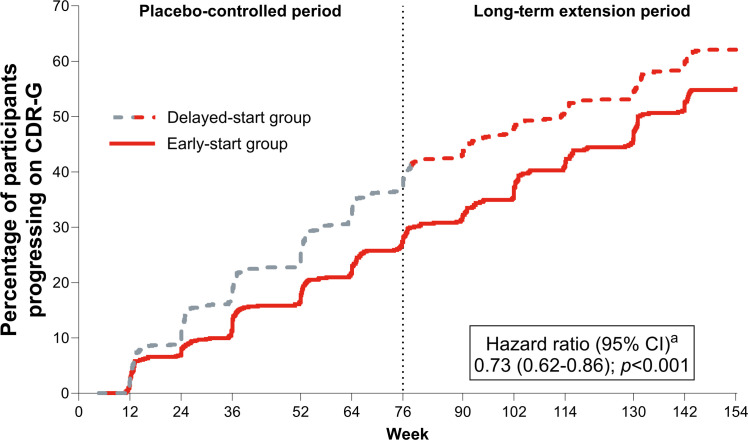
CDR-G hazard progression: early-start vs delayed-start treatment with donanemab. Early-start participants showed a 27 % reduced risk of progressing to the next clinical stage of the disease versus delayed-start participants. ^a^ Hazard ratio, 95 % CI, and p value were calculated using a Cox proportional hazards model. The model was stratified by pooled investigator and baseline tau level and included baseline covariates of age, CDR-G score, and acetylcholinesterase inhibitor/memantine use. CDR-G: Clinical Dementia Rating Scale–Global; CI: confidence interval. Copyright © 2025 Eli Lilly and Company. All rights reserved. Permission for any use should be sought from Eli Lilly and Company.

Donanemab treatment slowed disease progression among early-start participants, with an adjusted mean treatment difference of −1.2 points (95 % CI, −1.7 to −0.7) in the CDR-SB score between donanemab and the weighted external ADNI control cohort at 3 years (Fig. 2a). Similar efficacy results were observed for participants who met treatment course completion criteria by 52 weeks of the placebo-controlled period (Fig. 2c). In a supporting AUC analysis of the early-start group versus the ADNI cohort on change in the CDR-SB score, a superior cumulative benefit of donanemab treatment was shown among early-start participants during the LTE period (**Fig. S6**), with an average treatment difference of 0.97 points across 78 weeks (*p* < 0.001). In a comparison of the early- versus delayed-start groups using the CDR-G score (Fig. 3), early-start participants showed a 27 % reduced risk of progressing to the next clinical stage of the disease versus delayed-start participants (hazard ratio=0.73 [95 % CI, 0.62–0.86]; *p* < 0.001). Donanemab treatment also slowed disease progression among delayed-start participants, with an adjusted mean treatment difference of −0.8 points (95 % CI, −1.3 to −0.3) in the CDR-SB score 76 weeks after initiating donanemab versus the weighted external ADNI control cohort (Fig. 2b). At 3 years, early-start donanemab treatment saved approximately 6.9 months versus the ADNI cohort as measured by the CDR-SB score. At the end of the LTE and 1.5 years after starting donanemab, delayed-start treatment saved approximately 5.6 months on CDR-SB progression.

### 3.3 Amyloid PET

**Fig. 4:**
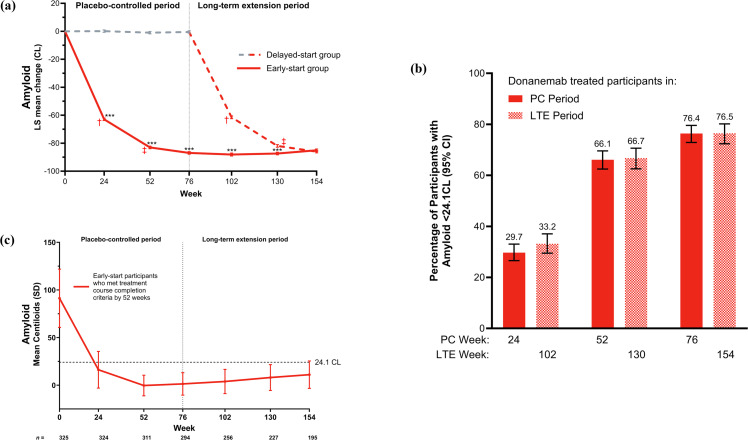
Brain amyloid plaque. The effect of donanemab on amyloid plaque was demonstrated by (a) mean reduction in amyloid over time between early-start and delayed-start treatment, (b) amyloid clearance in participants in the early-start and delayed-start groups at 24, 52, and 76 weeks after donanemab initiation, and (c) amyloid reaccumulation among participants in the early-start group who met treatment course completion criteria by 52 weeks of the placebo-controlled period. ***p < 0.001 vs delayed-start treatment; ^†^24 weeks from the start of donanemab treatment; ^‡^52 weeks from the start of donanemab treatment. CL: Centiloids; LTE: long-term extension; LS: least-squares; PC: placebo-controlled; SD: standard deviation; SE: standard error. Copyright © 2025 Eli Lilly and Company. All rights reserved. Permission for any use should be sought from Eli Lilly and Company.

Early-start participants achieved an LS mean (standard error) reduction in amyloid plaque of 86.96 (0.92) CL at 76 weeks. Delayed-start participants achieved a similar reduction (86.01 [0.89] CL) at 154 weeks, 76 weeks after initiating donanemab (Fig. 4a). After initiating donanemab treatment, amyloid clearance (<24.1 CL) was achieved by a similar percentage of early- and delayed-start participants at week 24 (29.7 % [*n* = 226/761] and 33.2 % [*n* = 196/591], respectively), week 52 (66.1 % [*n* = 443/670] and 66.7 % [*n* = 345/517], respectively), and week 76 (76.4 % [*n* = 469/614] and 76.5 % [*n* = 345/451], respectively) (Fig. 4b). Additional early-start participants (56.8 %) who continued donanemab treatment in the LTE period achieved amyloid clearance (<24.1 CL) assessed by an amyloid PET scan at the end of the LTE. **Table S3** presents the demographics and disease characteristics of early-start participants who did not achieve amyloid clearance (<24.1 CL) by the end of the LTE period (*N* = 33).

A subset of participants who achieved amyloid clearance (<24.1 CL) also met the trial treatment course completion criteria. For early-start participants with amyloid PET scans who continued donanemab treatment in the LTE, 29.9 % (*n* = 44/147) met treatment course completion criteria at 102 weeks, 45.6 % (*n* = 62/136) at 130 weeks, and 55.6 % (*n* = 70/126) at 154 weeks. For delayed-start participants with amyloid PET scans who received donanemab in the LTE, 17.6 % (*n* = 105/595) met treatment course completion criteria at 102 weeks (24 weeks after starting donanemab), 51.3 % (*n* = 267/520) at 130 weeks (52 weeks after starting donanemab), and 70.7 % (*n* = 319/451) at 154 weeks (76 weeks after starting donanemab). These values are similar to the percentage of donanemab-treated participants in the placebo-controlled period who met treatment course completion criteria at 24 weeks (17.1 %), 52 weeks (46.6 %), and 76 weeks (69.2 %). **Table S4** provides an overview of the numbers and percentages of early- and delayed-start participants who either achieved amyloid clearance or met treatment course completion criteria.

Early-start participants who met treatment course completion criteria by 52 weeks of the placebo-controlled period maintained a mean (SD) amyloid level of 10.99 (14.41) CL at 154 weeks, which is below the 24.1 CL cutoff for amyloid negativity (Fig. 4c). From data across four donanemab studies (including the results of this LTE), amyloid plaque reaccumulation was calculated to be 2.4 CL/year.

### 3.4 Safety

Safety data from the LTE period are shown in Table 1. The incidences of death, serious AEs, and TEAEs in delayed-start participants who received at least one infusion during the LTE were 1.1 %, 19.6 %, and 86.5 %, respectively. Two deaths during the LTE period were previously reported [13]; one was due to ARIA-E, and one was due to intracranial hemorrhage. The death due to intracranial hemorrhage occurred following thrombolytic administration in a participant where an MRI scan on the same day showed severe ARIA-E. The frequencies of infusion-related reaction (IRR), ARIA-E, and ARIA-microhemorrhages and hemosiderin deposits (ARIA-H) in the delayed-start group were 7.5 %, 26.0 %, and 39.7 %, respectively, during the LTE period. The event frequencies and overall safety profile of this group are comparable to the previously reported data for donanemab-treated participants during the placebo-controlled period [2].

**Table 1:** Safety overview and AEs of special interest during the LTE period.

Event, n ( %)	Early-start participants dosed in the LTE^a^		Delayed-start participants dosed in the LTE^a^
	Donanemab → placebo (N = 393)	Donanemab → donanemab (N = 157)	Placebo → donanemab (N = 657)
Overview			
Deaths^b^	8 (2.0 %)	2 (1.3 %)	7 (1.1 %)^c^
SAEs	80 (20.4 %)	21 (13.4 %)	129 (19.6 %)
Study discontinuations due to AE	15 (3.8 %)	6 (3.8 %)	39 (5.9 %)
Treatment discontinuations due to AE	15 (3.8 %)	6 (3.8 %)	89 (13.5 %)
TEAEs^d^	315 (80.2 %)	133 (84.7 %)	568 (86.5 %)
TEAEs deemed related to study treatment^e^	49 (12.5 %)	52 (33.1 %)	315 (47.9 %)
AEs of special interest			
ARIA-E	5 (1.3 %)	13 (8.3 %)	171 (26.0 %)
ARIA-H	24 (6.1 %)	30 (19.1 %)	161 (24.5 %)
Headache	15 (3.8 %)	11 (7.0 %)	65 (9.9 %)
Infusion-related reaction	2 (0.5 %)	11 (7.0 %)	49 (7.5 %)
Superficial siderosis of the CNS	4 (1.0 %)	6 (3.8 %)	44 (6.7 %)
Cerebral microhemorrhage	5 (1.3 %)	5 (3.2 %)	21 (3.2 %)
Vomiting	8 (2.0 %)	4 (2.5 %)	21 (3.2 %)
Nausea	6 (1.5 %)	5 (3.2 %)	17 (2.6 %)
ARIA overview			
Any ARIA^f^	59 (15.0 %)	54 (34.4 %)	289 (44.0 %)
Any SAE of ARIA^g^	0 (0.0 %)	1 (0.6 %)	9 (1.4 %)
ARIA-E^f^	6 (1.5 %)	13 (8.3 %)	171 (26.0 %)
Symptomatic ARIA-E	1 (0.3 %)	3 (1.9 %)	40 (6.1 %)
SAE of ARIA-E^g^	0 (0.0 %)	1 (0.6 %)	9 (1.4 %)
ARIA-H^f^	55 (14.0 %)	50 (31.8 %)	261 (39.7 %)
Symptomatic ARIA-H	1 (0.3 %)	0 (0.0 %)	3 (0.5 %)
SAE of ARIA-H^g^	0 (0.0 %)	0 (0.0 %)	0 (0.0 %)
Macrohemorrhage^f^	0 (0.0 %)	0 (0.0 %)	7 (1.1 %)
SAE of macrohemorrhage^g^	0 (0.0 %)	0 (0.0 %)	1 (0.2 %)

Note: Participants may be counted in more than one category.

AE: adverse event; ARIA: amyloid-related imaging abnormality; ARIA-E: amyloid-related imaging abnormality–edema/effusion; ARIA-H: amyloid-related imaging abnormality–microhemorrhages and hemosiderin deposits; CNS: central nervous system; LTE: long-term extension; MRI: magnetic resonance imaging; N: number of participants in the analysis population; n: number of participants with at least one event per type; SAE: serious adverse event; TEAE; treatment-emergent adverse event.

a Includes participants who received at least one infusion during the LTE.

b Deaths are also included as SAEs and discontinuations due to AEs.

c Includes two previously reported deaths due to ARIA-E and intracranial hemorrhage. Intracranial hemorrhage occurred following thrombolytic administration where an MRI scan on the same day showed severe ARIA-E [13].

d TEAEs are baseline AEs defined as all ongoing AEs at the first LTE dose; the postbaseline period started the day of the first LTE infusion and ended at the earlier date of study withdrawal or completion, the end of the LTE period + 57 days, or data cutoff.

e Includes events that were considered related to study treatment as judged by the investigator.

f Based on MRI or TEAE cluster.

g Based on TEAE cluster.

The incidences of death, serious AEs, and TEAEs in early-start participants who were switched to placebo infusions at or before the start of the LTE and received at least one placebo infusion during the LTE were 2.0 %, 20.4 %, and 80.2 %, respectively. The frequencies of IRR, ARIA-E, and ARIA-H in this group were 0.5 %, 1.5 %, and 14.0 %, respectively, during the LTE period. Among early-start participants who continued to receive donanemab treatment in the LTE period, the event frequencies during the LTE were generally less than those of donanemab-treated participants during the placebo-controlled period [2].

An analysis of the time to the first ARIA-E event among all participants exposed to donanemab (**Fig. S7**) revealed that most events occurred within the first 24 weeks of treatment. **Fig. S8** shows that the incidence of ARIA-E was 1.0 % or less in each 6-month interval following participants’ last dose of donanemab and that the risk of ARIA-H continued to decrease after the last dose of donanemab. The frequencies of symptomatic ARIA-E and symptomatic ARIA-H within any single 6-month period did not exceed 0.1 % and 0.3 %, respectively, after the last dose of donanemab. Among early-start participants who met treatment course completion criteria by 52 weeks based on amyloid reduction, the frequencies of any ARIA, ARIA-E, and ARIA-H after switching to placebo were 20.3 %, 2.0 %, and 18.3 %, respectively, with no ARIA-related serious AEs reported (**Table S5**). Following treatment course completion by 52 weeks, the OAIRs (rate adjusted by participant observation time) for early-start participants were similar to participants who received placebo during the placebo-controlled period (1.0 for ARIA-E and 10.9 for ARIA-H vs 1.5 for ARIA-E and 10.4 for ARIA-H, respectively).

## 4 Discussion

The results of this study provide further evidence that donanemab successfully modifies the course of early symptomatic AD with a growing treatment effect up to 3 years compared to an untreated population. Amyloid plaque reduction after treatment with donanemab was robust regardless of whether participants were in the early- or delayed-start treatment groups; however, overall efficacy favored participants who started early treatment with donanemab. The long-term benefits of donanemab were retained among participants who completed treatment within 1 year following successful amyloid clearance. After completing donanemab treatment, the rate of amyloid reaccumulation was comparable to the natural history of the disease [14,15].

The unique design of the TRAILBLAZER-ALZ 2 trial requires special consideration when interpreting its results. First, it is essential to note that the majority (71.5 %) of early-start participants who continued into the LTE received only blinded placebo infusions for the entire duration of the LTE period. Therefore, the observed efficacy of the early-start group reflects participants who experienced sustained efficacy of donanemab despite no longer receiving active treatment. Second, the delayed-start participants who entered the LTE also had more severe disease at the start of the LTE than the study population at the start of the placebo-controlled period [2]. In fact, almost half of the delayed-start participants would not have met the initial eligibility criteria for the study based on their MMSE score alone. Despite this, the delayed-start group also showed separation in the CDR-SB score from the ADNI control cohort. Finally, blinding was retained for both investigators and participants during the LTE period of TRAILBLAZER-ALZ 2, including once participants were switched to placebo infusions after successful amyloid reduction. This aspect of the design reduces the possibility that the results are attributed to participant or observer bias, as could be the case in a more traditional open-label extension design.

The efficacy results from the LTE provide further evidence that treating AD earlier on the disease continuum is more likely to result in better long-term outcomes. At 3 years, participants who were treated earlier with donanemab (ie, early-start group), as compared to participants who started donanemab treatment later (ie, delayed-start group), demonstrated a 27 % lower risk of progressing to the next stage of disease as assessed by the CDR-G score. This difference provides the best evidence of clinical meaningfulness from the available LTE data. Although it is tempting to compare the CDR-SB data from this study with the established values for minimal clinically important difference, these thresholds evaluate individual patient change and are not fit to interpret between-group differences at a study endpoint [16]. In contrast, any change on the CDR-G indicates a change in disease stage, which is inherently clinically meaningful. Additionally, estimated time savings at the end of the LTE as measured by the CDR-SB score were 6.9 months with early-start donanemab compared with 5.6 months following delayed-start treatment. These time savings represent the slowing of symptom progression in earlier stages of the disease, which is more impactful than the slowing of symptom progression in later stages of disease based on qualitative and quantitative research with both people living with AD and their care partners [17,18]. Importantly, the time saved by starting donanemab earlier may not necessarily come at the cost of extended time on treatment, given the limited-duration dosing approach with donanemab. Finally, although the absolute difference in time savings observed between early- and delayed-start donanemab is 1.3 months, the rate of disease progression is generally higher at later stages, which is not accounted for in our calculation. Time-based quantifications of treatment effect such as the progression model for repeated measures and the latent time-disease progression model have offered improvements to conventional quantifications in terms of meaningfulness, cumulative benefit summarization, post-trial implications, and cross-trial comparability [19]. Extrapolations of long-term efficacy scenarios suggest that donanemab may delay the onset of severe dementia by up to 0.6 years, 1.9 years, and 4.2 years with conservative, intermediate, and optimistic modeling assumptions, respectively [20]. Updated progression models that include data from the LTE period of TRAILBLAZER-ALZ 2 may provide additional insight into the time-based benefit of initiating donanemab earlier in the disease course.

The clinical efficacy of donanemab observed during the LTE period is supported by robust amyloid reduction, which was nearly identical between the early- and delayed-start groups at 76 weeks after donanemab treatment was initiated. The percentage of participants who achieved amyloid clearance was also strikingly similar between the early- and delayed-start participants at 76 weeks after initiating donanemab treatment, suggesting that the biologic effects of donanemab on amyloid plaque are similar in a population with further disease progression. When combined with data from previous donanemab studies, participants who met treatment course completion criteria by 52 weeks of the placebo-controlled period showed an amyloid reaccumulation rate (2.4 CL/year) comparable to the natural amyloid accumulation rate [14,15], reinforcing previously reported conclusions on slow plaque reaccumulation following donanemab treatment. Future analyses are needed to evaluate other AD-relevant biomarkers such as levels of phosphorylated tau protein using the plasma P-tau217 assay.

Details and characterizations of ARIA for the donanemab program were recently published [2]. The relationship between antidrug antibodies and IRRs has also been described [21]. Importantly, no new safety signals were observed during the LTE period compared to the established safety profile of donanemab. Safety observations up to 18 months after initiation of donanemab were consistent between participants in the early- and delayed-start groups, with similar frequencies of ARIA and IRR reported [2]. Participants who did not meet treatment course completion criteria, and thus remained on donanemab for more than 76 weeks, showed a consistent safety profile with data observed from donanemab-treated participants during the placebo-controlled period, with reduced frequencies of ARIA and IRR noted during the LTE. Of note, results from the TRAILBLAZER-ALZ 6 study (NCT05738486) recently showed that a more gradual dose titration of donanemab significantly reduced ARIA-E risk when compared with the standard dosing regimen utilized during TRAILBLAZER-ALZ 2, including in the LTE [22].

The LTE period of TRAILBLAZER-ALZ 2 is limited by the lack of a true placebo comparator and potential survivor bias over time. Use of an external control cohort may introduce differences in study conduct and assessments, time periods and geographic regions of data collection, and other potential unmeasured confounding factors. Inclusion criteria may limit the ability to generalize to broader clinical populations; however, the comorbidities and co-medications in the TRAILBLAZER-ALZ 2 population were consistent with the Medicare population [23]. Additional studies are ongoing to understand donanemab treatment in patients with varying stages of AD severity (eg, preclinical AD). Disease severity at the start of the LTE was more advanced in delayed-start participants than in early-start participants, likely because delayed-start participants did not receive treatment intervention during the 18-month placebo-controlled period. This difference may create biased efficacy results toward the early-start group when compared to the delayed-start group. All statistical analyses were exploratory and not controlled for multiplicity.

## 5 Conclusion

Over 3 years of observation, donanemab-treated participants with early symptomatic AD demonstrated increasing clinical benefits and a consistent safety profile with limited-duration dosing. These data support limited-duration dosing with treatment course completion based on amyloid reduction and reinforce the importance of intervention during the early stages of AD.

## Funding

This study was funded by 10.13039/100004312Eli Lilly and Company.

## Declaration of generative AI and AI-assisted technologies in the writing process

No generative AI or AI-assisted technologies were used in the preparation of this manuscript.

## CRediT authorship contribution statement

**Jennifer A. Zimmer:** Writing – review & editing, Investigation. **John R. Sims:** Writing – review & editing, Methodology, Investigation, Conceptualization. **Cynthia D. Evans:** Writing – review & editing, Investigation. **Emel Serap Monkul Nery:** Writing – review & editing, Investigation. **Hong Wang:** Writing – review & editing, Investigation. **Alette M. Wessels:** Writing – review & editing. **Giulia Tronchin:** Writing – review & editing. **Shoichiro Sato:** Writing – review & editing. **Lars Lau Raket:** Writing – review & editing, Methodology, Formal analysis. **Scott W. Andersen:** Writing – review & editing, Visualization, Formal analysis. **Christophe Sapin:** Writing – review & editing, Formal analysis. **Marie-Ange Paget:** Writing – review & editing, Formal analysis. **Ivelina Gueorguieva:** Writing – review & editing. **Paul Ardayfio:** Writing – review & editing. **Rashna Khanna:** Writing – review & editing. **Dawn A. Brooks:** Writing – review & editing, Supervision, Funding acquisition. **Brandy R. Matthews:** Writing – review & editing. **Mark A. Mintun:** Writing – review & editing, Methodology, Conceptualization.

## Declaration of interest

The authors declare the following financial interests/personal relationships which may be considered as potential competing interests:

Financial support was provided by 10.13039/100004312Eli Lilly and Company. Jennifer A. Zimmer, John R. Sims, Cynthia D. Evans, Emel Serap Monkul Nery, Hong Wang, Alette M. Wessels, Giulia Tronchin, Shoichiro Sato, Lars Lau Raket, Scott W. Andersen, Christophe Sapin, Marie-Ange Paget, Ivelina Gueorguieva, Paul Ardayfio, Rashna Khanna, Dawn A. Brooks, Brandy R. Matthews, and Mark A. Mintun report a relationship with Eli Lilly and Company that includes: employment and equity or stocks. Eli Lilly and Company has patents that are planned, issued, or pending related to this research.

## Appendix Supplementary materials

Image, application 1

## References

[1] Hardy J.A., Higgins G.A. (1992). Alzheimer's disease: the amyloid cascade hypothesis. Science.

[2] Sims J.R., Zimmer J.A., Evans C.D., Lu M., Ardayfio P., Sparks J. (2023). Donanemab in early symptomatic Alzheimer disease: the TRAILBLAZER-ALZ 2 randomized clinical trial. JAMA.

[3] van Dyck C.H., Swanson C.J., Aisen P., Bateman R.J., Chen C., Gee M. (2023). Lecanemab in early Alzheimer's disease. N Engl J Med.

[4] Roche Diagnostics (2023). Elecsys total-tau CSF. Method sheet.

[5] Gueorguieva I., Chow K., Chua L., Shcherbinin S., Zimmer J.A., Evans C.D. (2025). Donanemab exposure-response in early symptomatic Alzheimer's disease. Alzheimers Dement.

[6] Faria C.A., Alves H.V.D. (2015). Charchat-Fichman H. The most frequently used tests for assessing executive functions in aging. Dement Neuropsychol.

[7] Benedetto U., Head S.J., Angelini G.D., Blackstone E.H. (2018). Statistical primer: propensity score matching and its alternatives. Eur J Cardiothorac Surg.

[8] Stürmer T., Rothman K.J., Avorn J., Glynn R.J. (2010). Treatment effects in the presence of unmeasured confounding: dealing with observations in the tails of the propensity score distribution–a simulation study. Am J Epidemiol.

[9] Navitsky M., Joshi A.D., Kennedy I., Klunk W.E., Rowe C.C., Wong D.F. (2018). Standardization of amyloid quantitation with florbetapir standardized uptake value ratios to the Centiloid scale. Alzheimers Dement.

[10] Zeltzer E., Schonhaut D.R., Mundada N.S., Blazhenets G., Soleimani-Meigooni D.N., Cho H. (2025). Concordance between amyloid-PET quantification and real-world visual reads. JAMA Neurol.

[11] Shcherbinin S., Evans C.D., Lu M., Andersen S.W., Pontecorvo M.J., Willis B.A. (2022). Association of amyloid reduction after donanemab treatment with tau pathology and clinical outcomes: the TRAILBLAZER-ALZ randomized clinical trial. JAMA Neurol.

[12] Gueorguieva I., Willis B.A., Chua L., Chow K., Ernest C.S., Shcherbinin S. (2023). Donanemab population pharmacokinetics, amyloid plaque reduction, and safety in participants with Alzheimer's disease. Clin Pharmacol Ther.

[13] Zimmer J.A., Ardayfio P., Wang H., Khanna R., Evans C.D., Lu M. (2025). Amyloid-related imaging abnormalities with donanemab in early symptomatic Alzheimer disease: secondary analysis of the TRAILBLAZER-ALZ and ALZ 2 randomized clinical trials. JAMA Neurol.

[14] Jagust W.J., Landau S.M. (2021). Alzheimer's disease neuroimaging initiative. Temporal dynamics of β-amyloid accumulation in aging and Alzheimer disease. Neurology.

[15] Elhefnawy M.E., Patson N., Mouksassi S., Pillai G., Shcherbinin S., Chigutsa E. (2025). Quantifying natural amyloid plaque accumulation in the continuum of Alzheimer's disease using ADNI. J Pharmacokinet Pharmacodyn.

[16] Lansdall C.J., McDougall F., Butler L.M., Delmar P., Pross N., Qin S. (2023). Establishing clinically meaningful change on outcome assessments frequently used in trials of mild cognitive impairment due to Alzheimer's disease. J Prev Alzheimers Dis.

[17] DiBenedetti D.B., Slota C., Wronski S.L., Vradenburg G., Comer M., Callahan L.F. (2020). Assessing what matters most to patients with or at risk for Alzheimer's and care partners: a qualitative study evaluating symptoms, impacts, and outcomes. Alzheimers Res Ther.

[18] Hauber B., Paulsen R., Krasa H.B., Vradenburg G., Comer M., Callahan L.F. (2023). Assessing what matters to people affected by Alzheimer's disease: a quantitative analysis. Neurol Ther.

[19] Raket L.L. (2025). Time-based measures for quantifying disease modification in Alzheimer’s disease. Alzheimers Dement.

[20] Raket L.L., Cummings J., Moscoso A., Villain N., Schöll M. (2024). Scenarios for the long-term efficacy of amyloid-targeting therapies in the context of the natural history of Alzheimer's disease. Alzheimers Dement.

[21] Mullins G.R., Ardayfio P., Gueorguieva I., Anglin G., Bailey J., Chua L. (2025). Donanemab immunogenicity in participants with early symptomatic Alzheimer's disease. Alzheimers Dement.

[22] Wang H., Nery E.S.M., Ardayfio P., Khanna R., Svaldi D.O., Shcherbinin S. (2025). The effect of modified donanemab titration on amyloid-related imaging abnormalities with edema/effusions and amyloid reduction: 18-month results from TRAILBLAZER-ALZ 6. J Prev Alzheimers Dis.

[23] Klein E.G., Schroeder K., Wessels A.M., Phipps A., Japha M., Schilling T. (2024). How donanemab data address the coverage with evidence development questions. Alzheimers Dement.

