# Supplementary material for: Donanemab in early symptomatic Alzheimer’s disease: results from the TRAILBLAZER-ALZ 2 long-term extension
Source: J Prev Alzheimers Dis. 2025 Dec 1;13(2):100446. doi: 10.1016/j.tjpad.2025.100446 (PMC12869028; doi:10.1016/j.tjpad.2025.100446)
Supplement: Supplementary file 1 [file mmc1.pdf]

Supplementary Material

Table S1. Demographics and disease characteristics at the start of the LTE.

	Early-start participants dosed in the LTE[a]		Delayed-start participants dosed in the LTE[a]
	**Donanemab → placebo (*N*=393)**	**Donanemab → donanemab (*N*=157)**	**Placebo → donanemab (*N*=657)**
Female sex, *n* (%)	229 (58.3%)	80 (51.0%)	374 (56.9%)
Age, years, mean (SD)	76.3 (6.0)	72.0 (5.6)	74.9 (6.1)
APOE ε4 status, *n* (%)			
Carrier	249 (63.4%)	126 (80.3%)	472 (71.8%)
Heterozygous	204 (51.9%)	86 (54.8%)	352 (53.6%)
Homozygous	45 (11.5%)	40 (25.5%)	120 (18.3%)
Noncarrier	144 (36.6%)	31 (19.7%)	185 (28.2%)
AChEI and/or memantine use, *n* (%)	247 (62.8%)	101 (64.3%)	447 (68.0%)
MMSE total score,[b] mean (SD)	20.9 (5.4)	21.0 (5.3)	19.8 (5.5)
MMSE score category,[b] *n* (%)			
≥27	70 (17.8%)	20 (12.7%)	70 (10.7%)
20–26	171 (43.5%)	76 (48.4%)	290 (44.1%)
<20	152 (38.7%)	61 (38.9%)	297 (45.2%)
CDR-SB score, mean (SD)	5.1 (2.9)	5.1 (3.0)	5.7 (3.2)
CDR-G score, *n* (%)			
0.5	173 (44.0%)	74 (47.1%)	241 (36.7%)
1	169 (43.0%)	63 (40.1%)	310 (47.2%)
2	42 (10.7%)	17 (10.8%)	95 (14.5%)
Amyloid level by PET, Centiloids, mean (SD)			
Screening, Placebo-controlled period	94.8 (31.6)	122.0 (34.8)	102.3 (34.6)
Baseline, LTE period	3.7 (11.3)	37.5 (21.1)	102.5 (36.0)
Tau level by PET, SUVR, mean (SD)	1.7 (0.4)	1.8 (0.4)	1.7 (0.4)

Note: The number of participants with nonmissing data was used as the denominator for categorical data.

[a] Includes participants who received at least one infusion during the LTE.

[b] Last nonmissing MMSE score before or at the start of study treatment in the LTE.

AChEI: acetylcholinesterase inhibitor; AD: Alzheimer's disease; *APOE*: apolipoprotein E; CDR-G: Clinical Dementia Rating Scale–Global; CDR-SB: Clinical Dementia Rating Scale–Sum of Boxes; LTE: long-term extension; MMSE: Mini-Mental State Examination; *N*: number of participants in the analysis population; *n*: number of participants in the specified category; PET: positron emission tomography; SD: standard deviation; SUVR: standardized uptake value ratio.

Table S2. Demographics and disease characteristics at study baseline among participants who met treatment course completion criteria by 52 weeks of the placebo-controlled period.

	**Early-start participants dosed in the LTE who met treatment course completion criteria**
	**Donanemab → placebo by 52 weeks (*N*=325)**
Female sex, *n* (%)	198 (60.9%)
Age, years, mean (SD)	74.8 (6.1)
APOE ε4 status, *n* (%)	
Carrier	198 (60.9%)
Heterozygous	167 (51.4%)
Homozygous	31 (9.5%)
Noncarrier	126 (38.8%)
AChEI and/or memantine use, *n* (%)	200 (61.5%)
MMSE total score,[a] mean (SD)	23.9 (2.6)
MMSE score category,[a] *n* (%)	
≥27	62 (19.1%)
20–26	263 (80.9%)
<20	0 (0.0%)
CDR-SB score, mean (SD)	3.8 (2.1)
CDR-G score, *n* (%)	
0.5	199 (61.2%)
1	118 (36.3%)
2	7 (2.2%)
Amyloid level by PET, Centiloids,[a] mean (SD)	90.8 (30.6)
Tau level by PET, SUVR,[a] mean (SD)	1.3 (0.2)

[a] Based on screening data.

AChEI: acetylcholinesterase inhibitor; AD: Alzheimer's disease; *APOE*: apolipoprotein E; CDR-G: Clinical Dementia Rating Scale–Global; CDR-SB: Clinical Dementia Rating Scale–Sum of Boxes; LTE: long-term extension; MMSE: Mini-Mental State Examination; *N*: number of participants in the analysis population; *n*: number of participants in the specified category; PET: positron emission tomography; SD: standard deviation; SUVR: standardized uptake value ratio.

Table S3. Demographics and disease characteristics among early-start participants who did not achieve amyloid clearance by the end of the LTE.

	**Early-start participants who did not achieve amyloid clearance by the end of the LTE (*N*=33)**
Female sex, *n* (%)	17 (51.5%)
Age at screening, years, mean (SD)	68.1 (4.8)
APOE ε4 status, *n* (%)	
Carrier	29 (87.9%)
Heterozygous	19 (57.6%)
Homozygous	10 (30.3%)
Noncarrier	4 (12.1%)
AChEI and/or memantine use at study baseline, *n* (%)	19 (57.6%)
MMSE total score,[a] mean (SD)	23.5 (3.3)
MMSE score category at screening, *n* (%)	
≥27	5 (15.2%)
20–26	28 (84.8%)
CDR-SB score, mean (SD)	
Baseline	3.5 (2.1)
Week 154	5.9 (3.7)
CDR-G score at study baseline, *n* (%)	
0.5	24 (72.7%)
1	8 (24.2%)
2	1 (3.0%)
Amyloid level by PET, CL, mean (SD)	
Screening	127.0 (40.0)
Week 154	43.9 (19.0)
Tau level by PET, SUVR, at screening, mean (SD)	1.4 (0.2)

Note: The number of participants with non-missing data was used as the denominator for categorical data. Clearance was defined as achieving amyloid plaque levels <24.1 CL.

[a] Last nonmissing MMSE score before or at the start of study treatment.

AChEI: acetylcholinesterase inhibitor; AD: Alzheimer's disease; *APOE*: apolipoprotein E; CDR-G: Clinical Dementia Rating Scale–Global; CDR-SB: Clinical Dementia Rating Scale–Sum of Boxes; CL: Centiloids; LTE: long-term extension; MMSE: Mini-Mental State Examination; *N*: number of participants in the analysis population; *n*: number of participants in the specified category; PET: positron emission tomography; SD: standard deviation; SUVR: standardized uptake value ratio.

Table S4. Participants with a PET scan who met treatment course completion criteria or amyloid clearance.

Weeks after treatment initiation, % (*n/N*)	Early-start participants				Delayed-start participants			
	Treatment course completion			Amyloid clearance	Treatment course completion			Amyloid clearance
	<11 CL on single PET scan	<25 CL on two consecutive PET scans	Total	<24.1 CL	<11 CL on single PET scan	<25 CL on two consecutive PET scans	Total	<24.1 CL
24	17.1% (130/761)	0	17.1% (130/761)	29.7% (226/761)	17.6% (105/595)	0	17.6% (105/595)	33.2% (196/591)
52	44.5% (299/672)	2.1% (14/672)	46.6% (313/672)	66.1% (443/670)	48.7% (253/520)	2.7% (14/520)	51.3% (267/520)	66.7% (345/517)
76	58.9% (365/620)	10.3% (64/620)	69.2% (429/620)	76.4% (469/614)	62.3% (281/451)	8.4% (38/451)	70.7% (319/451)	76.5% (345/451)

Note: Data are shown as the percentage of participants with nonmissing values who met the criteria at the specified timepoint (number of participants who met criteria/total number of participants with a PET scan).

CL: Centiloids; *n*: number of participants who met criteria; *N*: total number of participants with a PET scan; PET: position emission tomography.

Table S5. ARIA overview after switching to placebo infusions among participants who met treatment course completion criteria by 52 weeks of the placebo-controlled period.

	Early-start participants dosed in the LTE who met treatment course completion criteria
**ARIA overview after switching to placebo infusions, *n* (%)**	**Donanemab → placebo by 52 weeks (*N*=306[a])**
Any ARIA[b]	62 (20.3%)
Any SAE of ARIA[c]	0 (0.0%)
ARIA-E[b]	6 (2.0%)
Symptomatic ARIA-E[d]	0 (0.0%)
SAE of ARIA-E[c]	0 (0.0%)
ARIA-H[b]	56 (18.3%)
Symptomatic ARIA-H[d]	1 (0.3%)
SAE of ARIA-H[c]	0 (0.0%)
Macrohemorrhage[b,e]	0 (0.0%)
SAE of macrohemorrhage[c]	0 (0.0%)

Note: Participants may be included in more than one category. Events with missing start date of post TEAE are excluded. Baseline is date of the first placebo infusion and observation time starts after the first placebo infusion.

[a] Includes participants who met treatment course completion criteria by 52 weeks and received at least one subsequent placebo infusion.

[b] Based on MRI or TEAE cluster.

[c] Based on TEAE cluster.

[d] Based on ARIA case report form for ARIA-E and adverse event reporting for ARIA-H.

[e] Macrohemorrhage includes cerebral hemorrhage and hemorrhagic stroke. The total ARIA counts do not include intracerebral hemorrhage.

ARIA: amyloid-related imaging abnormality; ARIA-E: amyloid-related imaging abnormality–edema/effusion; ARIA-H: amyloid-related imaging abnormality–microhemorrhages and hemosiderin deposits; LTE: long-term extension; MRI: magnetic resonance imaging; *N*: number of participants in the analysis population; *n*: number of participants with at least one event per type; SAE: serious adverse event; TEAE; treatment-emergent adverse event.

Fig. S1. Sensitivity analysis for clinical efficacy measured by change from baseline in the CDR-SB score for the early-start donanemab group versus external ADNI control cohort.

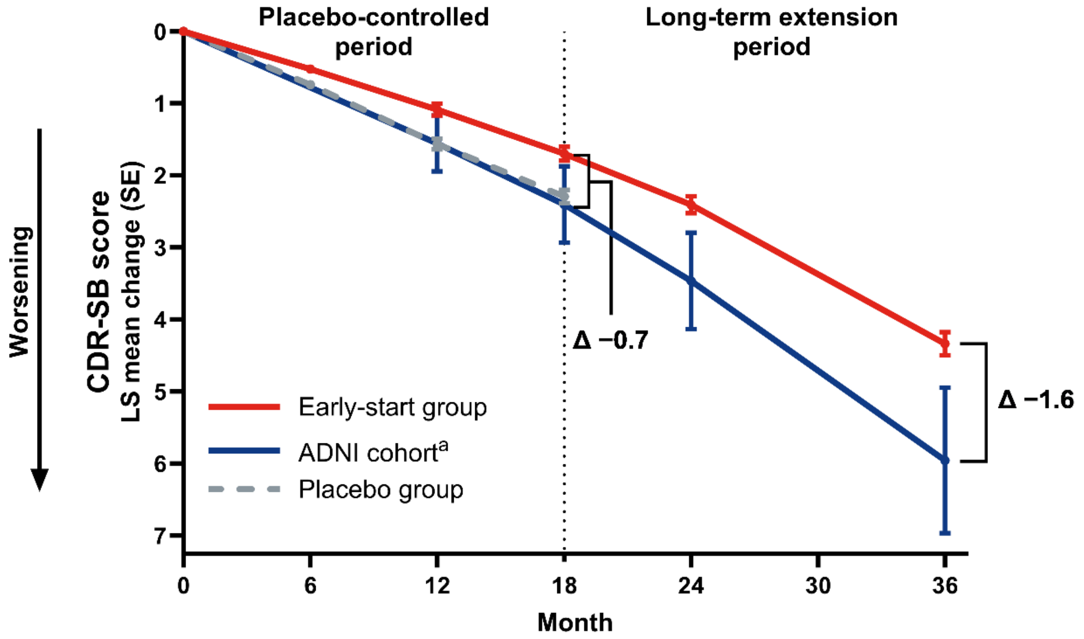

Early start vs ADNI difference:			-0.5	-0.7	-1.1		-1.6
Early-start group (*N*):	794	731	650	604	507		417
ADNI cohort[a] (*N*):	42		38	3	17		15[b]
Placebo group (*N*):	840	783	714	680			

Trajectories for early-start donanemab and placebo groups were estimated using a prespecified mixed model for repeated measures. Trajectories for the ADNI cohort were estimated using a random-coefficient spline model. Models were adjusted to identical baseline CDR-SB levels.

[a] Includes ADNI participants with amyloid pathology on PET (≥37 CL) and who were global tau PET positive.

[b] Includes nine observations beyond 36 months.

ADNI: Alzheimer's Disease Neuroimaging Initiative; CDR-SB: Clinical Dementia Rating Scale–Sum of Boxes; LS: least-squares; *N*: number of participants; PET: positron emission tomography; SE: standard error.

Fig. S2. Study disposition for the TRAILBLAZER-ALZ 2 LTE period.

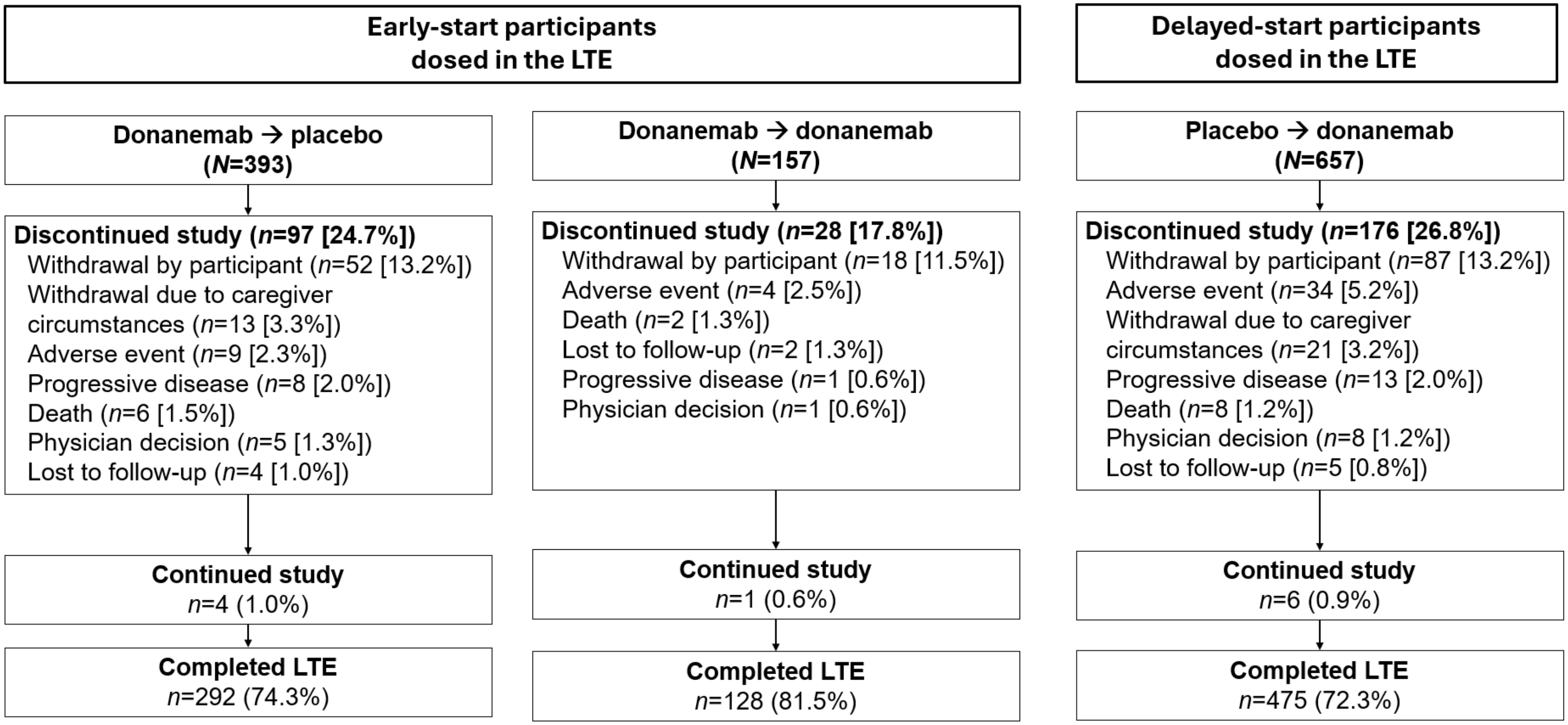

Disposition of the TRAILBLAZER-ALZ 2 LTE period includes all participants who received at least one infusion during the LTE.
LTE: long-term extension.

Fig. S3. Selection of the external ADNI control cohort for the (a) early-start and (b) delayed-start donanemab groups.

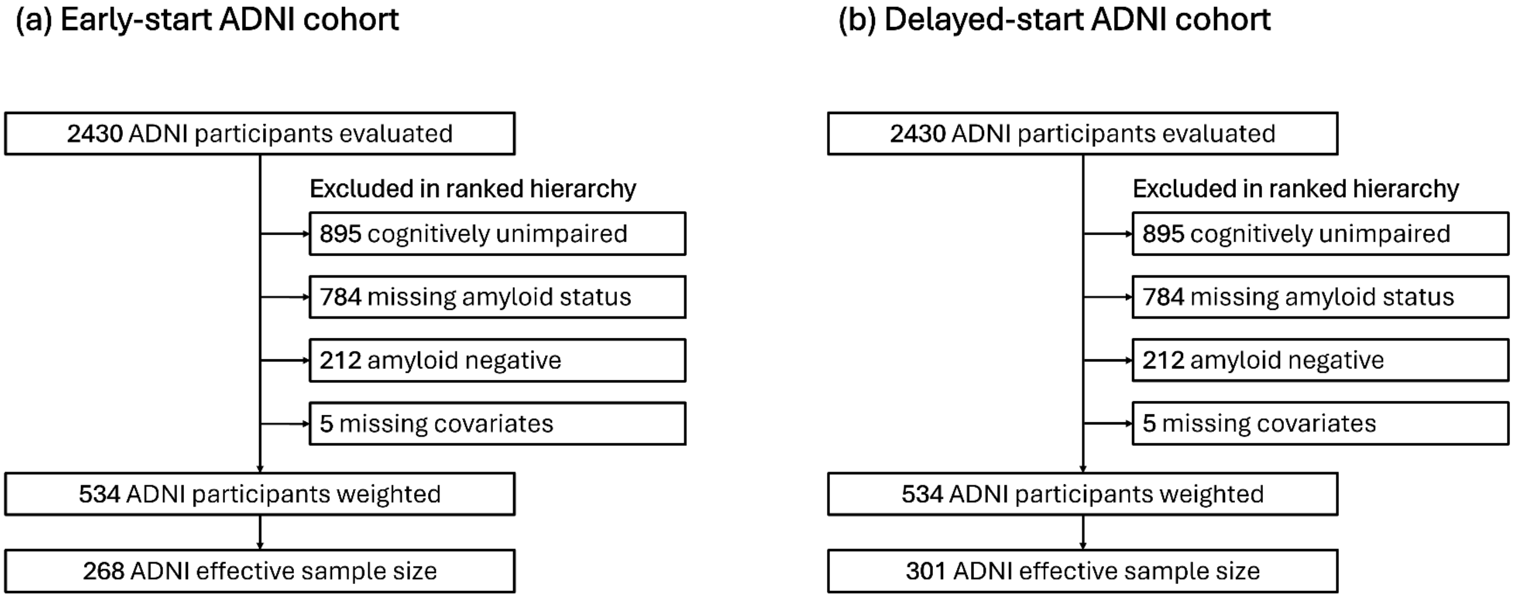

Participants were selected from the ADNI to meet the following clinical and biomarker criteria: presence of cognitive impairment and cerebrospinal fluid total tau-by-amyloid β42 ratio >0.28 (established criterion for amyloid pathology).
ADNI: Alzheimer's Disease Neuroimaging Initiative.

Fig. S4. Covariate balance of propensity weights in (a) early-start and (b) delayed-start donanemab groups.

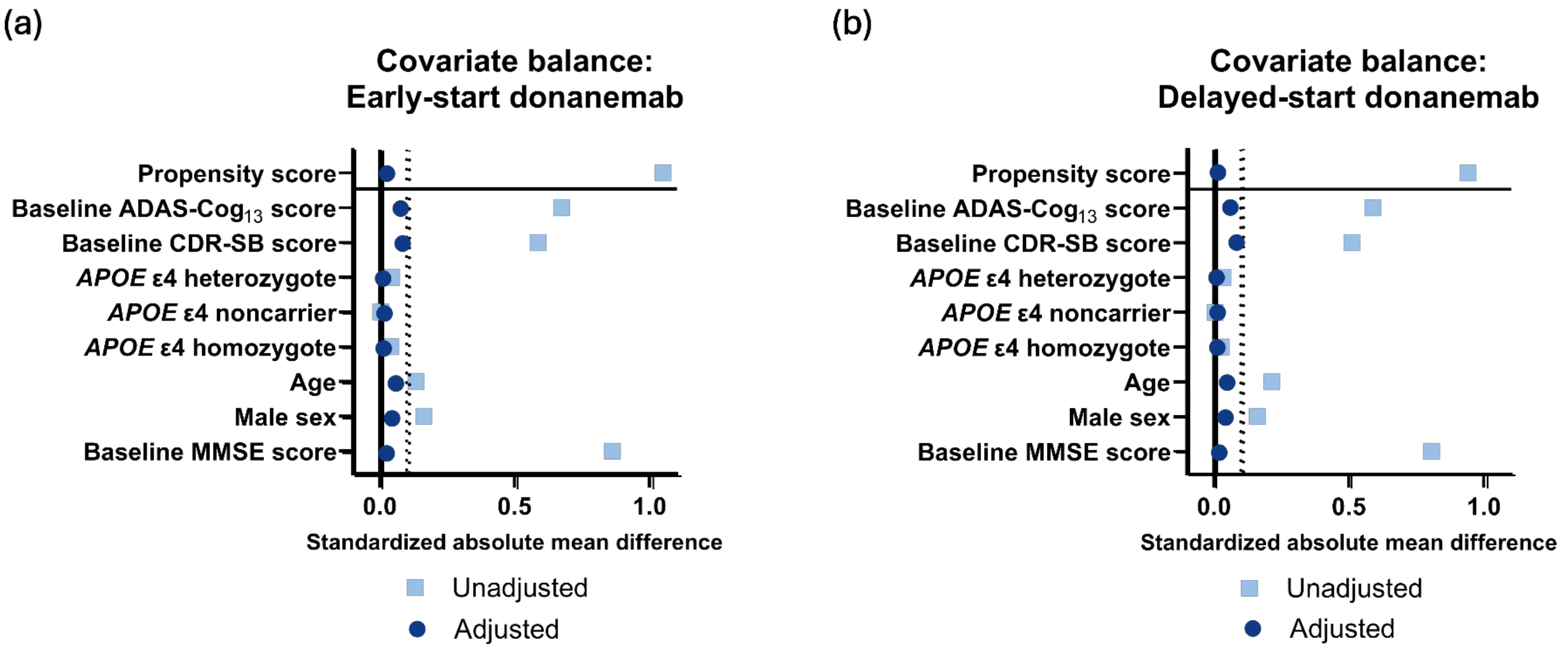

Propensity weights were checked to ensure balanced covariates for both the early-start and delayed-start groups. The vertical dotted line indicates a standardized absolute mean difference of 0.10.

ADAS-Cog$_{13}$: 13-item cognitive subscale of the Alzheimer Disease Assessment Scale; *APOE*: apolipoprotein E; CDR-SB: Clinical Dementia Rating Scale–Sum of Boxes; MMSE: Mini-Mental State Examination.

Fig. S5. Adjusted mean change in CDR-SB score for the placebo group versus the external ADNI control cohort.

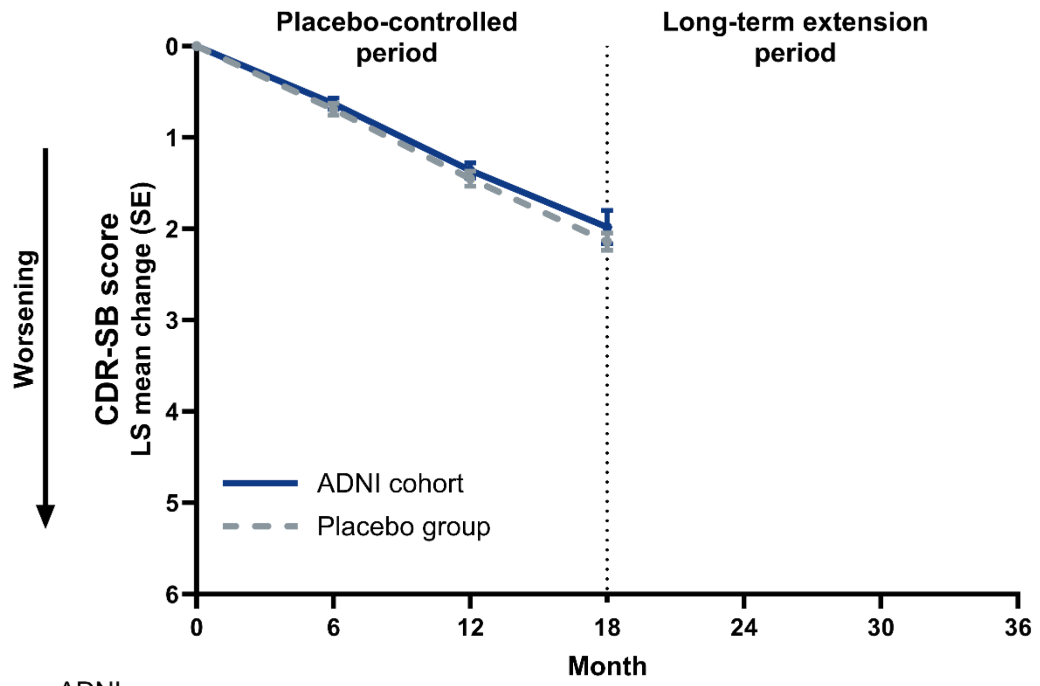

	0	6	12	18
Placebo vs ADNI difference (95% CI):		0.1 (-0.1, 0.2)	0.1 (-0.1, 0.3)	0.2 (-0.2, 0.5)
ADNI cohort (ESS):	268	255	237	200
Placebo group (*N*):	840	783	714	680

The external ADNI control cohort closely aligned with the trajectory of the placebo group, suggesting it is an appropriate estimate for the natural progression of Alzheimer's disease.
ADNI: Alzheimer's Disease Neuroimaging Initiative; CDR-SB: Clinical Dementia Rating Scale–Sum of Boxes; CI: confidence interval; ESS: effective sample size; LS: least-squares; *N*: number of participants; SE: standard error.

Fig. S6. Cumulative benefit of early-start donanemab treatment as shown by the adjusted mean change in CDR-SB score.

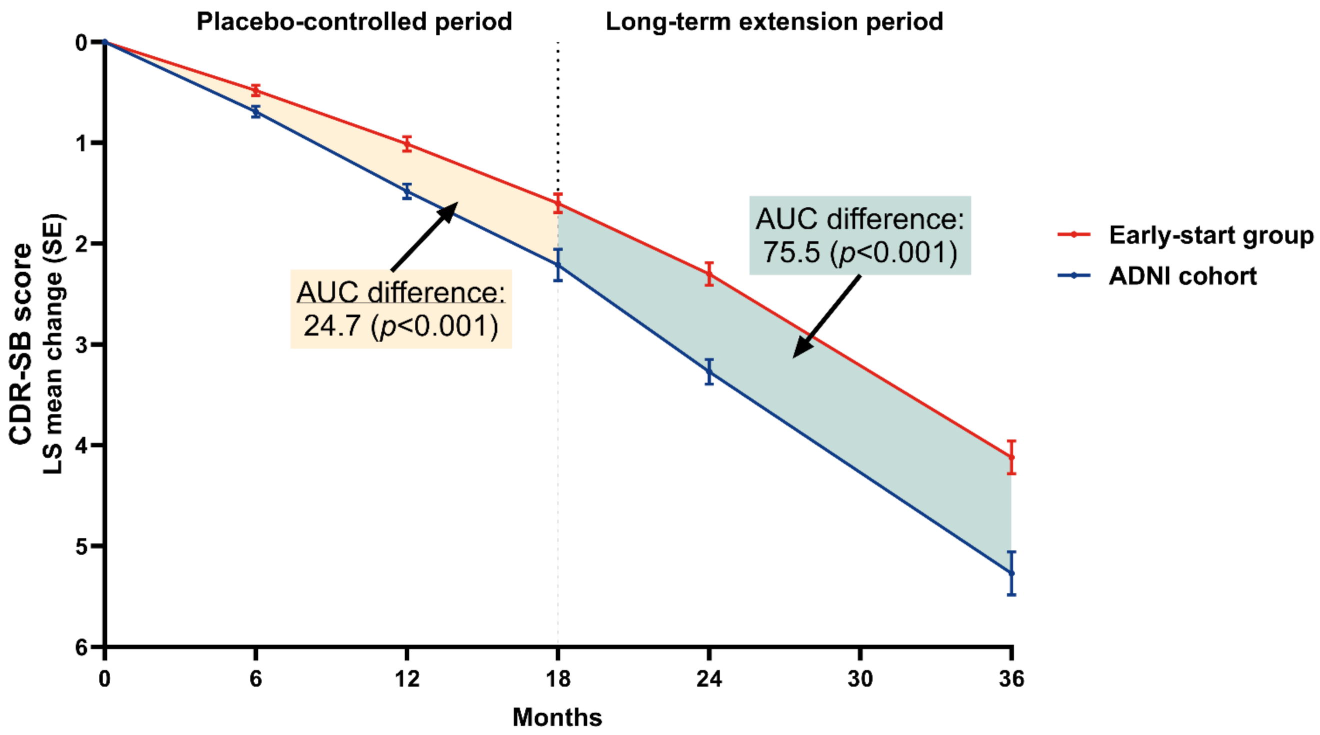

The AUC difference in CDR-SB score between early-start donanemab participants and the external ADNI control cohort continued to grow over time. Participants in the early-start donanemab group demonstrated an average treatment difference of 0.97 points in the CDR-SB score versus those in the external ADNI control cohort across the 78-week long-term extension period (*p*<0.001).

ADNI: Alzheimer's Disease Neuroimaging Initiative; AUC: area under the curve; CDR-SB: Clinical Dementia Rating Scale–Sum of Boxes; LS: least-squares; SE: standard error.

Fig. S7. Time to the first ARIA-E event.

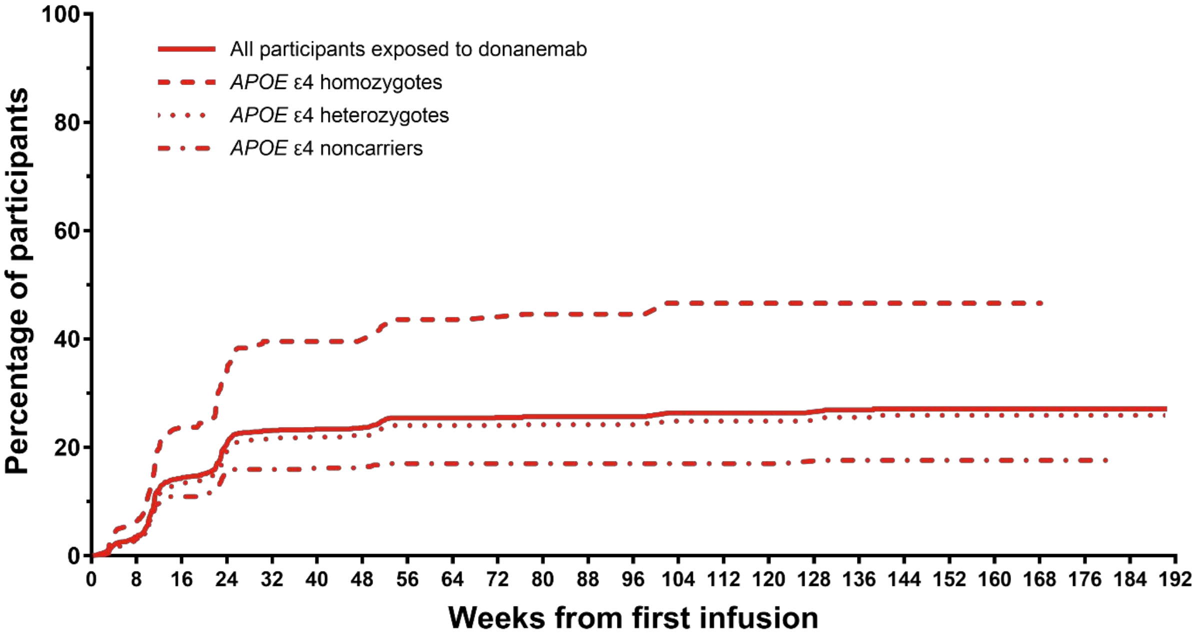

Number of participants at risk

All participants:	1510	1458	1281	1145	1083	1043	1004	957	923	891	823	491	450	429	419	409	398	383	371	362	318	26	3	1	0
Homozygotes:	263	247	199	169	146	143	135	122	116	113	106	60	56	53	51	51	49	47	45	44	37	2	0	0	0
Heterozygotes:	804	781	691	622	595	566	547	520	507	485	446	262	239	230	228	225	218	213	206	201	177	16	2	1	0
Noncarriers:	440	427	389	353	341	333	321	314	299	293	271	169	155	146	140	133	131	123	120	117	104	8	1	0	0

ARIA-E events in this analysis were based on magnetic resonance imaging or treatment-emergent adverse event cluster reporting. The timeframe for this analysis included the treatment period plus 57 days after the last donanemab dose.

APOE: apolipoprotein E; ARIA-E: amyloid-related imaging abnormality–edema/effusion.

Fig. S8. Summary of ARIA after the last donanemab dose.

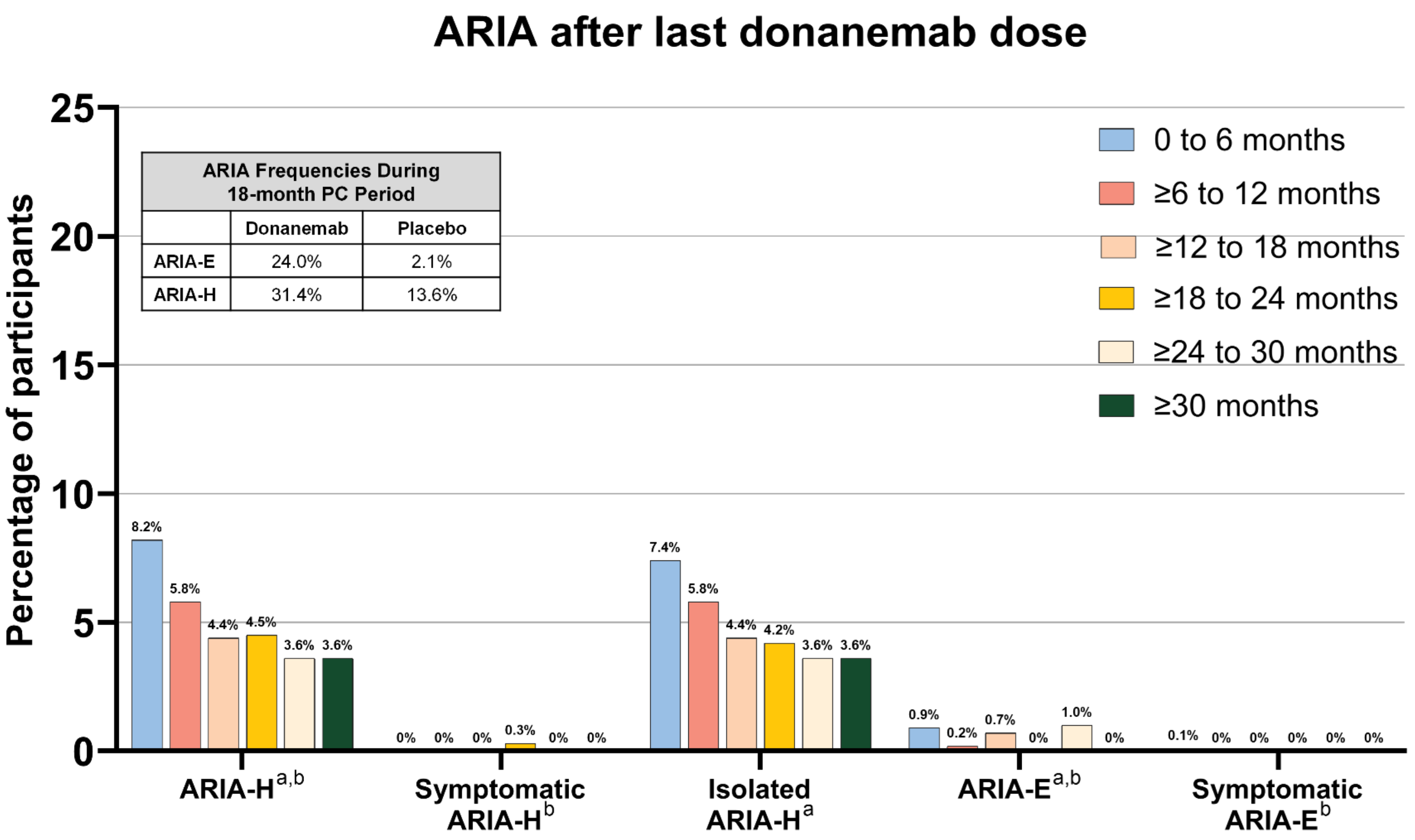

The first time interval (0–6 months) started at 58 days after the last dose of donanemab.

[a] New events identified by magnetic resonance imaging.

[b] New or worsening (compared to the endpoint of the previous interval) events identified by adverse event data.

ARIA: amyloid-related imaging abnormality; ARIA-E: amyloid-related imaging abnormality–edema/effusion; ARIA-H: amyloid-related imaging abnormality–microhemorrhages and hemosiderin deposits; PC: placebo-controlled.
